# An agent-based simulation combined with group decision-making technique for improving the performance of an emergency department

**DOI:** 10.1590/1414-431X20175955

**Published:** 2017-03-30

**Authors:** M. Yousefi, R.P.M. Ferreira

**Affiliations:** Departamento de Engenharia Mecânica, Universidade Federal de Minas Gerais, Belo Horizonte, MG, Brasil

**Keywords:** Agent-based simulation, Patient flow, Decision making, Emergency department, Health industry

## Abstract

This study presents an agent-based simulation modeling in an emergency department. In a traditional approach, a supervisor (or a manager) allocates the resources (receptionist, nurses, doctors, etc.) to different sections based on personal experience or by using decision-support tools. In this study, each staff agent took part in the process of allocating resources based on their observation in their respective sections, which gave the system the advantage of utilizing all the available human resources during the workday by being allocated to a different section. In this simulation, unlike previous studies, all staff agents took part in the decision-making process to re-allocate the resources in the emergency department. The simulation modeled the behavior of patients, receptionists, triage nurses, emergency room nurses and doctors. Patients were able to decide whether to stay in the system or leave the department at any stage of treatment. In order to evaluate the performance of this approach, 6 different scenarios were introduced. In each scenario, various key performance indicators were investigated before and after applying the group decision-making. The outputs of each simulation were number of deaths, number of patients who leave the emergency department without being attended, length of stay, waiting time and total number of discharged patients from the emergency department. Applying the self-organizing approach in the simulation showed an average of 12.7 and 14.4% decrease in total waiting time and number of patients who left without being seen, respectively. The results showed an average increase of 11.5% in total number of discharged patients from emergency department.

## Introduction

Emergency departments (EDs) operate 24 h a day, 7 days a week, and their high operating cost is a cause for budget shortages. The staff needs to attend any patient who arrives and to provide them the best possible service. More importantly, EDs deal with human health. Therefore, an error in ED procedures may lead to disability or death. In general, public hospitals operate under several constraints. In some cases, these constraints are incompatible with each other. Patients arrive at EDs with different health issues and different levels of severity and treatment is provided according to these aspects, i.e., emergency, semi-emergency or non-emergency. Depending on the country, different models are used to categorize the patients in EDs. Although the structure of EDs can vary from one country to another, they all have some characteristics in common. All EDs have resources such as physicians, nurses, receptionists, technicians, beds, and medical and laboratory equipment. The flow process of patients is as follow: registration, triage, laboratory/X-ray exams, placement in a specific section in the ED, and treatment. At the end of the process, patients can be discharged or transferred to the hospital for further treatment.

EDs have high costs and limited resources. Therefore, some vital decisions must be made accurately to avoid wasting time and money, and eventually providing low quality treatment. For complex systems such as EDs, there is no standard model to help organize the performance of the system and a high risk of using the trial and error method exists. Consequently, simulation methods can become the main technique to organize EDs without disrupting its routine. Several studies show that approximately 50% of all hospital admissions are initiated in ED. Therefore, the main entrance of patients to hospitals are through EDs (1).

Selecting a key performance indicator (KPI) for simulation of EDs is a controversial subject. Although there is no rule-of-thumb to select a KPI, the most commonly used in this field of study are the number of patients who leave the ED without being seen (LWBS), number of discharged patients, length of stay (LOS), time lapse for seeing a doctor, and average waiting times.

## Material and Methods

As one of the most important elements in the health industry, EDs get the attention of researchers in various aspects in order to provide a tool to help hospital managers improve the efficiency and effectiveness of these departments (2). The main objectives of studies on EDs are to improve the quality of service using quality management concepts, reducing patients’ waiting time and to study the complexity of EDs using computer models, multi-criteria decision-making models and optimization methods (3).

Application of computer simulation in the health care industry to improve staff scheduling goes back to the late 1970s (4). Several studies focused on solving the overcrowding problem in EDs using simulation techniques (5–8).

Dawson et al. (9) presented a theoretical model for the UK National Health Service to investigate the impact of expanding patient choice on waiting time. The study of Samaha et al. (10) tried to reduce the length of stay in EDs by presenting a simulation model of operations in an ED. Another study in Finland presented a model to investigate the impact of a novel triage method on waiting times and the number of discharged patients from EDs (11). Ahmed and Alkhamis (12) provided a simulation optimization method in an ED in Kuwait to reduce the waiting times and increase patient’s throughput. Their model led to a 40% reduction in waiting time and 28% increase in patient's throughput. Several studies also presented combined models to reduce waiting times in EDs. For instance, Laskowski et al. (13) applied a queuing model and an agent-based simulation model to reduce waiting times.

Three main simulation approaches are vastly applied in the literature of the healthcare industry: discrete event simulation (DES) (14), agent-based simulation (ABS) (15), and system dynamics (SD) (16). Although DES is the preferred method, recent reviews show that from 2011 the use of ABS in EDs simulation has been increasing (17). DES is process-oriented and has a rich availability of software, thus has been applied in many cases. However, interacting decision-making abilities of ABS make it a more reliable tool.

### Agent-based model of EDs

This paper proposes a pure agent-based simulation for EDs. The model was formed based on information obtained from the relevant literature. The information about the ED process was extracted from a DES simulation study (12). In order to add behavior variables of patients, technicians, nurses and doctors to the model, numerous human behaviors in EDs were extracted from literature.

### Structural model

**Figure 1:**
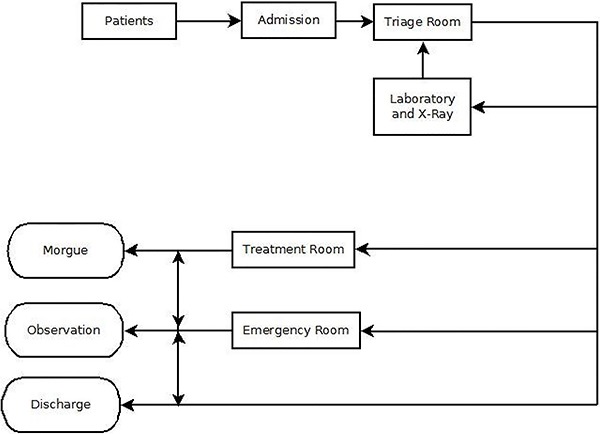
Simplified flow of patients in an emergency department.

Figure 1 demonstrates the flow of patients in an ED in the UK. Patients arrive to the ED waiting room 1 by either walk-in, with an ambulance or in police custody. Then, they wait to be registered by a receptionist, who collects the patients' information including name, age, etc. The patient then goes to the waiting room 2 and waits for the availability of the triage room. In the triage room a nurse (or a doctor) checks the severity of the patient based on the Manchester Triage System (MTS) (18). The MTS contains various flowcharts that categorize patients into five different priority categories: red, orange, yellow, green and blue, being red the highest priority and blue the lowest priority level.

Fifty percent of patients need laboratory or x-ray exams to be categorized. Patients with blue level are treated in the triage room and discharged. Patients with yellow and green levels that need minor procedures receive treatment in the treatment room by a nurse, and patients with orange and red levels are assigned to a bed in an emergency room and treated by a nurse and a doctor. Eighty-eight percent of patients are discharged from the emergency room, while the remainder are admitted to the hospital for further treatment.

The arrival process of patients to the waiting room 1 is a non-homogenous Poisson process (Figure S1). The λ(t) stands for the estimated function of patient arrival per hour. In the triage room, nurses assign almost 33% of patients to a wrong category. A total of 7.6% of all errors is over-triage and 25.3% is under-triage. For more details, see (19). As examples, under-triage refers to patients who should be in the category orange but a nurse triages them in the category yellow, and over-triage occurs when the patient should be in the yellow category, but the triage nurse triages them in the green category. Errors in over-triage can lead to patients being sent to a wrong section of the service and there the staff of that section redirects the patient to the right section. In under-triage, the error may not be discovered and the patient will be discharged without receiving proper treatment. In this model, they are recognized as wrongly discharged patients.

In every step of the process, patients can decide to continue the treatment or leave the ED without being treated. The model assumes that patients with red and orange priority levels would not leave the ED without receiving care, because they would be in a serious condition and be prioritized to receive treatment. Fifty-one percent of patients wait up to 2 h in the ED to receive treatment, 17% wait 2 to 8 h, and the rest (32%) wait indefinitely for treatment (20).

According to the MTS, all patients must receive treatment within a specific amount of time. To add this parameter to the model, each patient (agent) has a specific number that is linked to their assigned priority. This number is extracted from APACHE II (Acute Physiology and Chronic Health Evaluation II), which is used by the Brazilian Ministry of Health as a criterion for classifying intensive care units (21). APACHE II was originally used in Intensive Care Units (ICUs) and its accuracy has been confirmed in several studies. For instance, Chiavone and Sens (21) evaluated the stratification of APACHEII in the ICU of Santa Casa de São Paulo Hospital from July 1998 to June 1999, showing that higher APACHE II scores are correlated to higher mortality rates.

In our model, the chance of dying was calculated based on their APACHE II number. If a patient waited longer times than what was specified, their APACHE II changed. Consequently, their mortality probability increases with increasing waiting time. If the APACHE II of a patient reached 30, the mortality probability was almost equal to 1. Each service in the ED needs a specific amount of time to be provided. Time distribution for each service was extracted from Ahmed and Alkhamis (12).

### Agents behavior

In ABS modeling, agents play the main role. Agents are able to interact with their environment as well as with other agents, evolving over time, which allows them to react to different situations in the system. The behavior of active agents (in our case patients, doctors, technicians, nurses) is modeled by a Moore state machine, which is a finite-state machine. Agents will stay in their current state until they interact with other agents and receive a new input. Each input is the output of another agent. In Moore state machine, the next state is a function of the current state and its inputs. Hence, the Moore state machine can be described as follows:

(1) X(n+1)f(x(n), i)

where, X(n) and X(n+1) are the state at the time n and state at the time n+1, respectively, and i stands for inputs. A Moore machine also can be descried by a 6 tuple (A, B, C, D, E, F) where A is a set of states, B is set of inputs, and C is set of outputs. D is the input transition function, where

(2) D:A×→A

and E is the output transition function, where

(3) E:A×B→C

F is the initial state from where any input is processed (F ε A).

In the transition, the state machine of the agent goes to the next state (S_t_+1), which can be the same as S_t_ or different. In other words, the input is described as vector (I), which is a set of variables with different values for each. In the Moore machine, each output is only dependent on the state; therefore, different states have their own output. It is possible that different states have the same output. In the same manner as the input, the output can be described as an output vector (O), which is a series of output variables. Each of these series has a number of defined possible values. The transition between states depends on two factors: input at time t (I_t_) and the current state at the time t (S_t_). In simple systems, the model is deterministic.

EDs are too complex and dynamic systems to be modeled with a totally deterministic model. In order to give the dynamicity to the model, more than one possibility for the next stage is given to the combination of current state and input. Therefore, the transition will be chosen randomly when an agent reaches the transition time. Each transition has a different possibility, which are given by different weights. For instance, we have an agent with a current state of S_x_ that receives an input I_a_. The agent may stay in the same state as before (S_x_), go to state S_y_ and to state S_z_. These states have different probability of P_1_, P_2_ and P_3_. One or more of these have to happen, therefore, P_1_ + P_2_ + P_3_ = 1. The same approach is used in ([Bibr B22]–24). Table 1 shows the different tasks that each agent can do during the simulation in ED. Agents might do all or only part of their activities in a simulation.

**Table 1:**
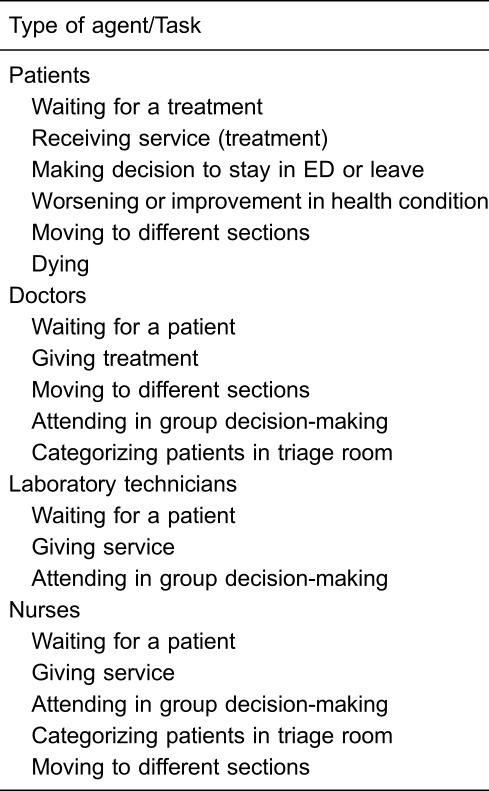
Types of agents and their activities in an emergency department (ED).

**Figure 2:**
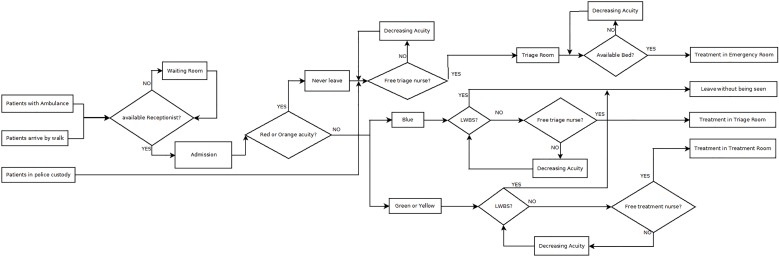
Flow of patients in an emergency department when the patients are categorized based on Manchester Triage System and they can leave without being seen (LWBS).

Figure 2 shows the detailed flow of patients in the emergency department based on their level of severity.

### Decision-making methodology

Decision-making tools are used in different studies related to simulation in emergency departments. For instance, Eskandari et al. (25) used multi-criteria decision-making methods such as analytic hierarchy process (AHP) and the Technique for Order of Preference by Similarity to Ideal Solution (TOPSIS) with respect to 9 different factors to choose the best scenario out of 14.

In this paper, a novel approach was applied in order to improve the performance of an ED. When there was more than one decision-maker with different attitudes, knowledge and ideas, they tended to reach a common solution for a problem (26). The proposed group decision-making (GDM) (27) was used in order to re-organize the staff in the ED based on different criteria. In this process, the total number of staff and their function remained without change. The GDM helped to re-allocate the resources during the process without its interruption. For instance, in case of an increase in number of patients waiting for triage and a decrease in number of patients waiting for treatment room, a decision was made to send one of the nurses from the treatment room to help in the triage room.

**Figure 3:**
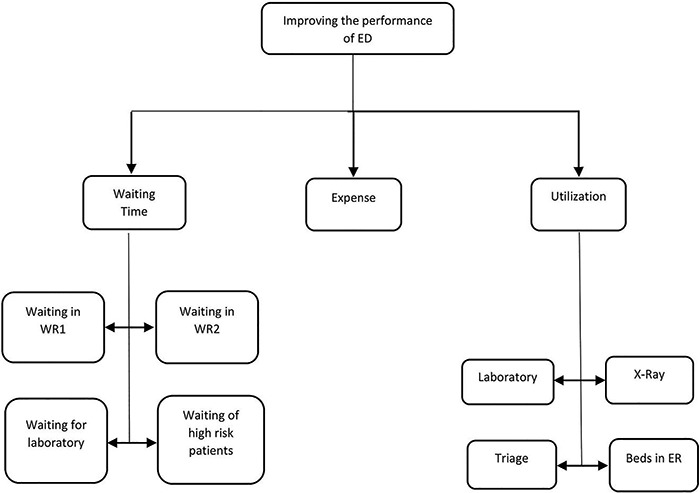
Criteria to use group decision making to improve performance of emergency department (ED). WR1: waiting room 1; WR2: waiting room 2; ER: emergency room.

Consider a situation in an ED in which a prompt decision is needed to improve the performance. There are many decision-makers (doctors, nurses and technicians) ***D*1**, …, ***Dl***, n scenarios (human resources in each section) ***A***1****,…,***A*n** and m different criteria ***A*1**,…,***A*m** (waiting time, length of stay, etc.). In this case, $a_{ij}^{k}$ is the result of the staff assessment for scenario ***A***i****, considering criteria ***C***i****. Different decision-makers might have different preferences on different criteria. It should be noted that the preference of a decision-maker (weight) ***D***k**** at criteria ***C***i****,***i*=1**,…,***m*;*k*=1**,…,***l*** is always a positive number $w_{i}^{k}\geq 0$. Decision-makers give different weights to criteria based on their knowledge and work in EDs, and also give different scores to each scenario (voting power). Figure 3 demonstrates the criteria collected in 3 main groups and eight sub-groups.

The voting power of ***Dk*** for weighing on criterion ***Ci*** is shown with $V{\left(w\right)}_{i}^{k}$. In the same manner, the voting power of ***Dk*** for scoring on criterion ***Ci*** shown as $v{\left(q\right)}_{i}^{k}$, where ***i***=1,…,***m*** and ***k***=1,…,***l*.** The group score for scenario ***Aj*** is calculated as follow:

First, the individual preference for each criterion ***Ci*** is aggregated into group weights ***Wi***:

(4) $$W_{i}=\frac{\sum_{k=1}^{l}V{\left(W\right)}_{i}^{k}W_{i}^{k}}{\sum_{k=1}^{l}V{\left(W\right)}_{i}^{k}},\;\;\;i=1,\ldots ,m$$

Then, the group score ***Qij*** of scenario ***Ai*** based on criterion ***Ci*** is calculated as follow:

(5) $$Q_{ij}=\frac{\sum_{k=1}^{l}V{\left(q\right)}_{i}^{k}a_{ij}^{k}}{\sum_{k=1}^{l}V{\left(q\right)}_{i}^{k}},\;\;\;i=1,\ldots ,m,j=1\ldots ,n$$

The weighted mean of the aggregated qualification values with the aggregated weights is the group utility ***Uj*** of ***Aj***, that is as follow:

(6) $$U_{j}=\frac{\sum_{i=1}^{m}W_{i}Q_{ij}}{\sum_{i=1}^{m}W_{i}},\;\;\;j=1,\ldots ,n$$

The resources of this ED cannot exceed the following limits: 3 receptionists, 12 emergency room nurses, 4 doctors (triage and emergency room), 6 nurses (triage and treatment room) and 5 lab technicians. The needed budget for each resource is as follows: 0.4 budget units (BU) for each receptionist, 1.2 BU for each doctor, 0.5 BU for each lab technician and 0.3 for each nurse (12).

### Communication between agents

One of the ABS features is the possibility of communication between agents. In fact, communication is an output that an agent produces and an input that another agent receives. There are three types of communication in ABS models. One occurs between two individual agents, which means that the message has one receiver and one sender. This type of communication is called one-to-one. For instance, the communication between a nurse and a patient in a triage room is a one-to-one. The other type of communication in ABS models is the communication of an individual agent with a group of agents. For instance, when a receptionist gives information to patients in registration the communication is a one-to-n. One-to-location is the third type of communication, which occurs when an agent is communicating with all agents in a specific area, for example, when a nurse sends a message to all patients in the waiting room (22,23).

### Computer simulation

**Figure 4:**
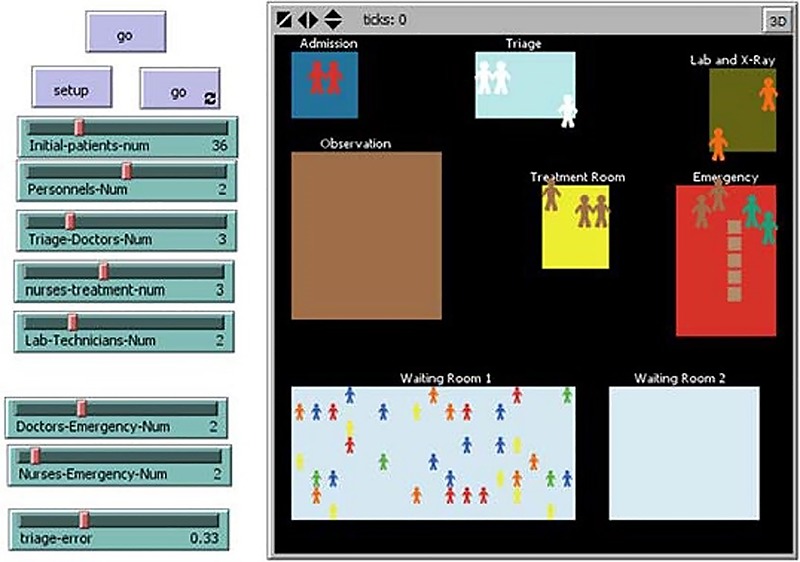
Graphical user interface and 2D visualization of the simulation in NetLogo software (https://ccl.northwestern.edu/netlogo/).

The proposed ABS model was designed and created using Netlogo (an open source simulation tool provided by Northwestern's Center for Connected Learning and Computer-based Modeling in Illinois, USA) (28), a high-level platform that is suited for multi-agent simulations dealing with hundreds of agents. Netlogo can simulate complex systems considering the micro-level behavior of each single agent and the macro-level that is the result of interactions of many agents with different characteristics. Netlogo also provides 2D and 3D visualization of all the simulated systems that show all the actions and interactions of different agents in each step of the process, which makes it a useful tool to get feedback from experts in the healthcare industry that have no experience in simulation. Figure 4 shows the user interface of the simulated model. As can be seen, the number of different variables is represented with slide bars through a graphical user interface. Therefore, the user can easily change the number of different element such as number of initial patients, number of personnel who work as receptionists, the number of nurses or doctors, the number of technicians and the probability of having a mistake in the triage room. Only those variables that the user needs to change are shown in the user interface, the others can be seen only in code.

One of useful features of Netlogo is a tool that allows users to perform experiments with models. In this paper, the BehaviorSpace tool is used to study the behavior of the system in different conditions.

### Description of scenarios

In order to study the flow of patients in an ED and its influence on self-organization ability of staff on the ED's performance, different scenarios were introduced and the performance of the ED in each scenario was observed. Table 2 shows the characteristics of different scenarios that were selected regarding all constraints of the study and the budget limitation.

**Table 2:**
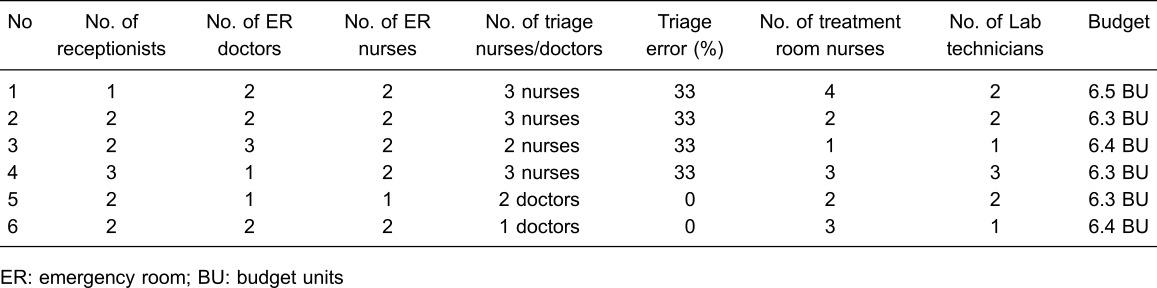
Characteristics of selected experiments.

For selecting scenarios, we tried to cover the accepted range for each variable, while the selected budget range was >6.2 and ≤6.5 BU. Scenarios with smaller budget amounts were excluded because their results were far from the baseline case study.

In scenario 4, the model tried to reduce the waiting time in waiting room 1 as much as possible with 3 receptionists, while in scenario 1, with 3 triage nurses and 4 treatment room nurses, the focus was more on reducing waiting time in waiting room 2. In 4 out of 6 scenarios, triage nurses were in charge of triaging, which means 33% of all patients will be assigned wrongly to a category. In scenario 5 and 6, the triage process was done by doctors, therefore we assumed that the triage error in these two scenarios was zero.

In this study, some of the most common KPIs were selected. The following is a short introduction on each of them.

*LWBS*: Patients that leave the ED when they find long queues and have to wait longer than what they can tolerate. Although they leave the system, they partially use the resources of EDs, since most leave the hospital after being registered or even after being triaged. Therefore, reducing their number is important for management.

*LOS*. Total time that a patient spends in an ED and begins by his or her arrival and ends when the patient leaves. Reducing mean LOS is key to improve the performance of EDs and increase patient satisfaction.

*Total waiting time*. Time that a patient waits to receive any service or treatment.

*Number of deaths*. Improving the performance of an ED helps to provide treatment for high-risk patients in a short period of time. In this model, the chance of dying for high-risk patients constantly increased while they did not receive treatment. The model assumed that the only cause of death in high-risk patients was long waiting time.

*Wrongly discharged*. Number of patients who are assigned to the wrong sector and discharged before receiving the proper treatment.

*Discharged patients*. Total number of discharged patients, which shows the throughput of the system. Management always tries to improve this indicator.

## Results

**Figure 5:**
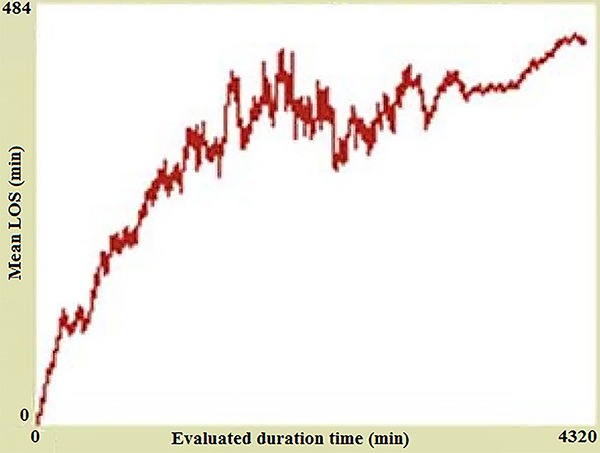
Fluctuation of length of stay (LOS) in warm-up period.

At the beginning of the simulation, the ED was empty; therefore the results would reflect the reality. To manage this problem, a warm-up period should be considered: the simulation ran for a while before it started to collect data. Figure 5 demonstrates the average LOS from time zero, when the ED was empty. As can be seen, after two days the graph reached a stable point. Therefore, 2 days of warm-up period was selected for this study. The simulation was run for 3 days (4,320 min) but the data collection began after 2 days (2,880 min) of simulation, which was what was used in the analysis.

Each scenario ran once without self-organization, meaning that the number of staff in each section was fixed until the end of simulation. When having self-organization, the staff could change their section based on the result of group decision-making. Therefore, the numbers in Table 2 are initial number of people in each section, which changed with time, in order to improve the patients’ experience in ED.

For each scenario, the simulation ran 200 times: 100 times without self-organization and 100 times with self-organization. The results are summarized in Table 3 and show that in all 6 scenarios there was an improvement in KPI.

**Table 3:**
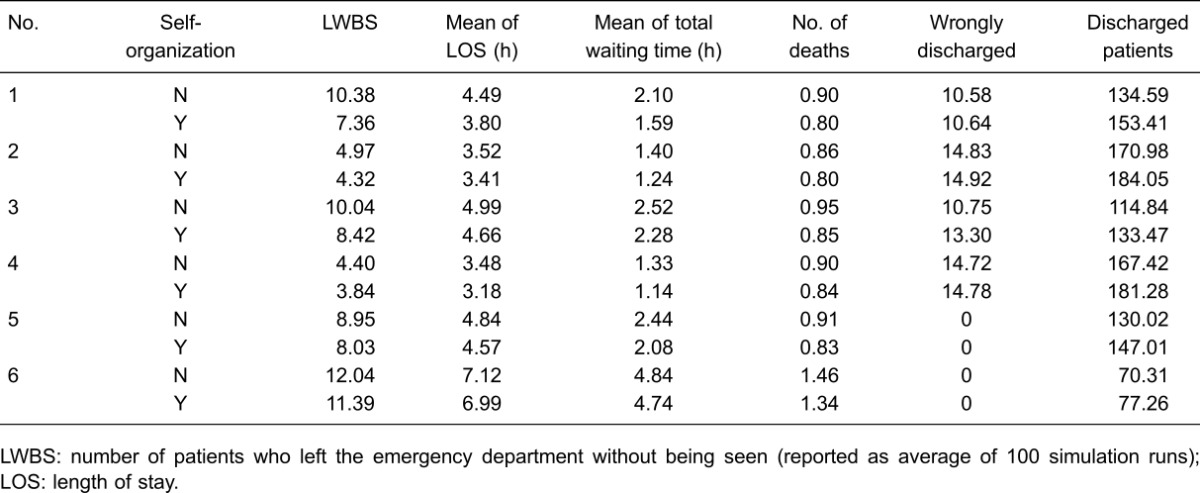
Measured key performance indicators for each scenario, with (Y) and without (N) group decision-making.

**Figure 6:**
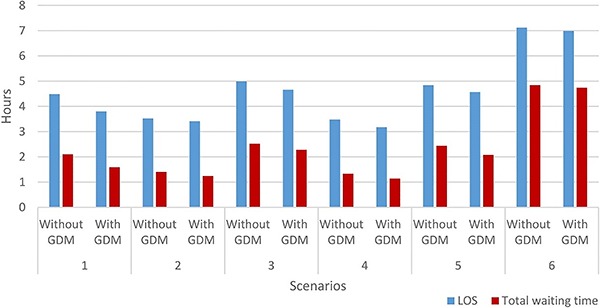
Comparison of average length of stay (LOS) and average total waiting time before and after applying group decision-making (GDM) in different scenarios.

Figure 6 demonstrates the improvement in LOS and waiting time after applying the group decision-making, without adding any extra resources. However, each scenario improved at different rates. Scenario 1 had a 15.3% improvement in average LOS and 24.2% improvement in total waiting time, showing the biggest reaction to the new approach. The total average improvement for LOS and waiting time were 6.8% and 12.7%, respectively.

**Figure 7:**
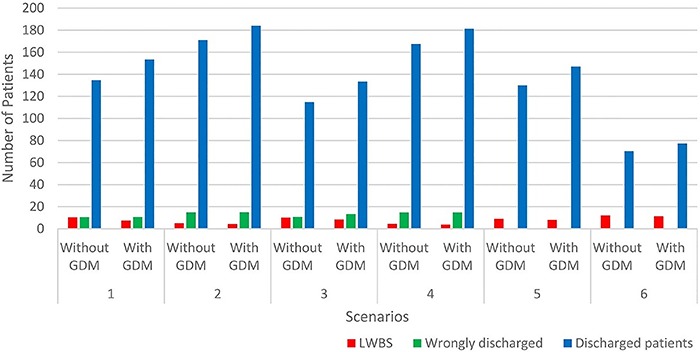
Comparison of leaving without being seen (LWBS), number of wrongly discharged patients and total number of discharged patients before and after applying group decision-making (GDM) in different scenarios.

Figure 7 exhibits the results for the other KPIs including LWBS, number of patients who were incorrectly discharged and number of patients that received treatment and left the ED. Although number of deaths is one of the parameters, its results are not discussed here, as the variation in number of deaths was negligible, as can be seen in Table 3.

There was no error in triage in scenarios 5 and 6, as we assumed that when the triage is done by a doctor, instead of a nurse, there will be no errors and all patients will be directed to the right section. Results did not show improvement in patients who were wrongly discharged, because this factor was not correlated with LOS or total waiting time nor with resource allocation. There was 14.4 and 11.5% average improvement for number of LWBS and discharged patients, respectively. Again, scenario 1 had the highest increase in number of discharged patients (29.1%) and LWBS (13.9%).

## Discussion

This study provides a general framework for agent-based simulation of emergency departments (EDs) based on their different characteristics. The behavior of each type of agent was extracted from the literature and from personal observation. The agents not only have the ability to make individual decisions but also are able to communicate with other agents and participate in group-decision making to improve the performance of the ED. The proposed agent-based simulation and group decision-making method can be easily implemented in any emergency department after some modifications.

In future studies, the same approach should be applied in a real-case scenario. Then, simulation results can be compared with the real data, after implementing group-decision making. The approach can also be applied on different types of problems in EDs.

## Supplementary Material

Click here to view [pdf].
